# Thymoma in Ectopic Thymus: An Overlooked Differential and a Pathologist’s Dilemma

**DOI:** 10.7759/cureus.94195

**Published:** 2025-10-09

**Authors:** Pranjal Kalita, Alexandra K Mawlong, Neoky Suiam, Mainak Bhattacharjee, Vandana Raphael

**Affiliations:** 1 Pathology, North Eastern Indira Gandhi Regional Institute of Health and Medical Sciences, Shillong, IND

**Keywords:** ectopic thymus, fine needle aspiration cytology (fnac), myasthenia gravis, thymoma, type b2 thymoma

## Abstract

Thymomas arising in thymic tissue located in an abnormal location are rarely described in medical literature and present with a wide range of diagnostic dilemmas. Amongst the abnormal locations for ectopic thymus, the thyroid serves as the most common site; however, case reports of ectopic thymic tissue have also been seen in rarer locations, such as the middle ear, base of the skull, submandibular gland, and tonsils. Myasthenia gravis (MG), a paraneoplastic syndrome associated with thymomas, is very rare in cases of ectopic site thymomas. In this report, we present one such case of a 44-year-old female patient who initially presented with generalised weakness, difficulty breathing, and loss of appetite for one week. Differential diagnoses of tuberculosis, lymphoma, and metastatic malignancies were considered clinically. A contrast-enhanced computed tomography of the neck, thorax, and abdomen indicated the presence of a mild homogenously enhancing mediastinal mass (6.6x5.4x4.0 cm) in the right para-tracheal region abutting the left distal brachio-cephalic vein and the superior vena cava. Prompt laboratory examination, specifically fine needle aspiration cytology and cell block evaluation of the mediastinal mass, helped categorise the lesion as type B2 thymoma. The patient was advised of a thymectomy but refused and presented months afterwards with a diagnosis of MG. Ectopic thymic foci along with the thymus were removed, and a diagnosis of type B2 thymoma in the mediastinal mass was substantiated. The reported case highlights the rarity of MG in patients with ectopic thymoma and the importance of a strong clinical suspicion and laboratory findings in making a correct diagnosis, eventually leading to better patient management.

## Introduction

Thymomas are rare tumors originating from the thymic epithelial cells, representing a meager 0.2-1.5% of all malignancies, with an estimated incidence of 0.13-0.32 per 100,000 annually [1]. Amongst the ectopic sites, the anterior (pre-vascular) mediastinum is the commonest location for ectopic thymic tissue presenting as thymoma. However, other sites, namely the middle (visceral), posterior (paravertebral) mediastinum, neck, pleura, lung, pericardium, and thyroid gland, may also serve as rare locations for ectopic thymomas [2].

Wu et al. reviewed the clinical data of 114 published cases of thymomas arising in ectopic sites in 2019, and noted a wide variation with respect to the age of presentation, gender predilection, size, site, and type of thymomas [2]. Unfortunately, such tumors are frequently misdiagnosed as other lesions that are more commonly located at these sites [3]. Diagnostic modalities, namely fine needle aspiration cytology (FNAC) or incisional biopsies, play a pivotal role in the proper categorization of such rare neoplasms in ectopic sites, even in the absence of a proper clinical suspicion. Thus, an awareness of the presence of ectopic thymomas and the potential diagnostic pitfalls is of utmost importance in accurate diagnosis and patient management, bearing prognostic implications. We present the case of a 44-year-old woman with myasthenia gravis (MG), eventually diagnosed with an ectopic thymoma that was initially overlooked in the differential diagnosis clinically.

## Case presentation

A 44-year-old female patient hailing from a remote village in north-east India presented to the emergency department with generalized weakness, difficulty in breathing, and a loss of appetite for one week. The discharge summary brought with her from a previous hospital admission in her village stated the presence of multiple enlarged lymph nodes in the pre- and para-tracheal region. On further questioning, it was revealed that her husband was diagnosed with pulmonary tuberculosis and had completed treatment one year prior.

**Figure 1:**
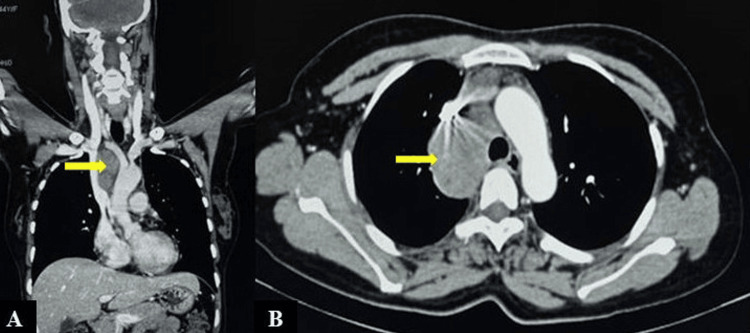
CT of neck, thorax and abdomen (A- Coronal view, B- Axial view) shows a mild homogenously enhancing mediastinal mass (6.6x5.4x4.0 cm) in right paratracheal region abutting the left distal brachio-cephalic vein and superior vena cava, shown by yellow arrows

Routine investigations revealed a haemoglobin level of 10.8 g% (reference range: 12-18 g%) with a total leukocyte count of 4x10^3^/ml (reference range: 4-11x10^3^/ml). A contrast-enhanced computed tomography (CECT) of the neck, thorax, and abdomen indicated the presence of a mild homogenously enhancing mediastinal mass, measuring 6.6x5.4x4.0 cm, in the right para-tracheal region abutting the left distal brachio-cephalic vein and the superior vena cava (Figure 1).

At this point, differential diagnoses of tuberculosis, lymphoma, and metastatic malignancies were considered clinically.

**Figure 2:**
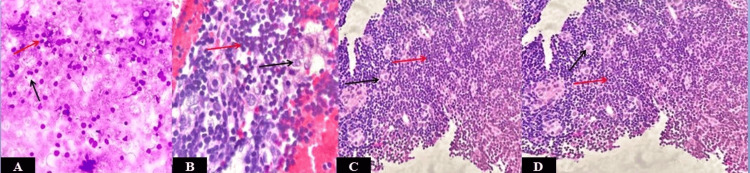
(A) FNAC showing two populations of cells, predominantly small to medium lymphoid cells and another population of medium to large epithelial cells (MGG, 200x). (B,C) Cell block showing small lymphocytes and epithelial cells with vesicular nucleus and moderate amount of cytoplasm (H&E, 200x). (D) Biopsy from right paratracheal mass confirming the presence of small lymphocytes and epithelial cells (H&E, 200x). Red arrows highlight lymphoid cells and black arrows highlight epithelial cells. MGG: May-Grünwald-Giemsa; H&E: hematoxylin and eosin

An FNAC from this lesion was advised and yielded moderately cellular smears with two distinct populations of cells. One was composed predominantly of small to medium lymphoid cells, while the other consisted of medium to large-sized epithelial cells (Figure 2A). Subsequent examination of the cell block showed small lymphocytes and larger epithelial cells with a vesicular nucleus and a moderate amount of cytoplasm, suggesting a possibility of ‘type B2 thymoma' (Figures 2B, 2C). Cytomorphological evaluation of type B2 thymoma presents with its inherent difficulty, considering the presence of two populations of cells, the lymphoid and epithelial cells. An immunohistochemistry study plays a key role in the diagnosis and ruling out other differential diagnoses. Pan cytokeratin (Pan Ck) positivity in the epithelial cells and positivity for terminal deoxynucleotidyl transferase (TdT) and cluster of differentiation-3 (CD3) in the immature T-lymphocytes supported the thymic origin. Absence of caseating granulomatous lesion ruled out tuberculosis, positivity for Pan CK ruled out lymphoma, and based on the clinical and radiological (CECT) findings, a possibility of metastasis was ruled out. Following this, a biopsy from the right para-tracheal mass was obtained, which confirmed the diagnosis of thymoma type B2 (Figure 2D).

**Figure 3:**
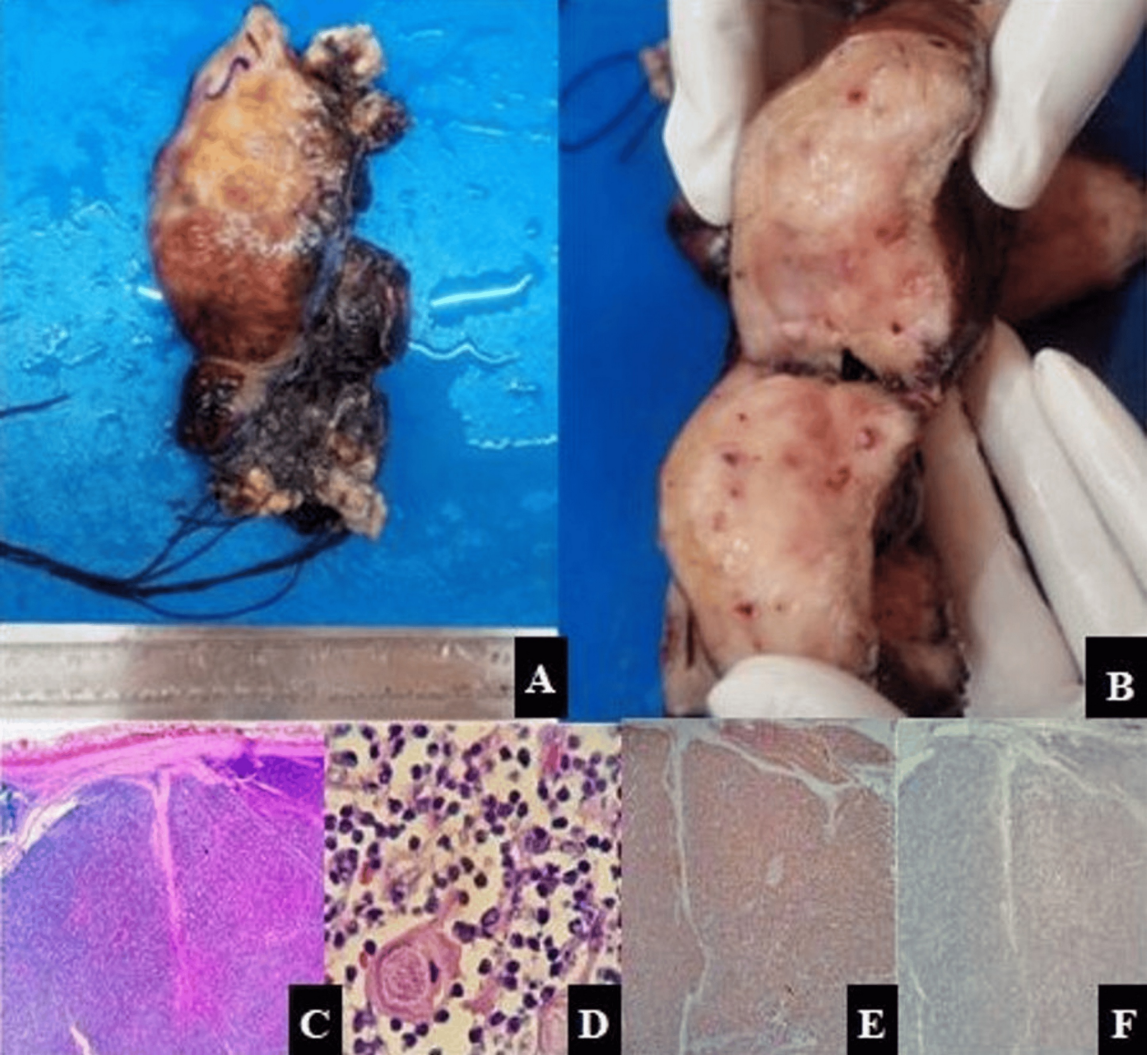
Gross examination reveals (A) an encapsulated soft tissue mass with a smooth outer surface and (B) a lobulated grey-tan appearance on cut section. (C) Histopathology of the resection specimen showing fibrous capsule with tumor cells arranged in lobules separated by fibrous septa (H&E, 100x) and (D) medullary islands (Hassall corpuscle-like elements, paler areas), scattered neoplastic epithelial cells, and immature T-lymphocytes (H&E, 400x). (E) Immunohistochemistry showing PanCK positive neoplastic epithelial cells (IHC, 200x) and (F) CD 117 negative ruling out thymic carcinoma (IHC, 200x) H&E: hematoxylin and eosin; IHC: immunohistochemistry

The patient was advised thymectomy, but at the time, she decided to return to her village first. She eventually returned to the hospital six months later due to the exacerbation of her symptoms, which included progressive worsening of generalized weakness, difficulty breathing, cough, and weakness of her arms and legs. It was learned that she had received a diagnosis of MG during the last six months. A neurology consultation was sought to confirm her diagnosis. She was prescribed pyridostigmine 60 mg thrice daily and azathioprine 50 mg once daily. Two months later, an open thymectomy was performed, and the thymus, along with the ectopic thymus, was removed (Figures 3A, 3B). The tumor was located entirely in the mediastinum and was anatomically distinct from the orthotopic thymus, showing no continuity on imaging or intraoperative examination.

Grossly, two specimens were received labelled as "Anterior mediastinal mass" and "Thymus". The anterior mediastinal mass specimen was an encapsulated, well-circumscribed, and lobulated soft tissue mass measuring 6.6 × 5.4 × 4.0 cm. The external surface was smooth in appearance with focal areas of congestion. On serial sectioning, the cut surface was solid, grey-white, and lobulated with focal areas showing fibrous septa. No areas of necrosis or calcification were identified grossly. The thymus specimen showed two lobes of thymus measuring 2.8 × 2.5 × 2.3 cm and 3.0 × 2.1 × 2.1 cm, respectively, and showed an unremarkable external surface. On serial sectioning, the cut surface showed a lobulated surface with fibrous septa and tan-pink parenchyma; no nodules, hemorrhagic, or necrotic areas were identified grossly.

Histopathological examination of the anterior mediastinal mass revealed a fibrous capsule with tumor cells arranged in lobules separated by fibrous septa, scattered medullary islands (Hassall corpuscle-like elements, paler areas), and neoplastic cells (Figure 3C, 3D). Neoplastic epithelial cells are polygonal in shape with vesicular nuclei, small, prominent nucleoli, and pale cytoplasm, admixed with abundant immature T lymphocytes. No areas of necrosis or atypical mitosis were noted. The resected margin for the mass was free of tumor. No capsular, vascular, or perineural invasion was noted. Histomorphology of the thymus specimen showed physiological thymic involution.

Immunohistochemistry with Pan CK, p40 immunostains was positive in the neoplastic epithelial cells. TdT and CD3 positivity were noted in immature T-lymphocytes, while CD117 and CD5 were negative, thus ruling out thymic carcinoma (Figures 3E, 3F). Ki-67 index in the neoplastic epithelial cells was 1%.

Based on the microscopic findings, the tumor was staged as T1aN0M0 (TNM staging). No extracapsular extension or nodal involvement noted. Considering the fact that the patient had a complete surgical resection (R0 resection), negative margin status, no aggressive histology, or any invasion, no adjuvant therapy was indicated. The postoperative hospital course was uneventful, and the patient was asked to follow up two months post discharge in the outpatient department. A CECT thorax at six months after discharge was also planned. No clinico-radiological recurrence was noted on follow-up visits as per available medical reports.

## Discussion

The thymus is a multi-lobulated and encapsulated organ derived from the third or fourth pharyngeal pouches, which descend into the anterior mediastinum in the sixth week of human gestation. The ectopic thymus is recognised as unencapsulated lobules of thymus or microscopic foci of thymic tissue found at locations other than its normal position, resulting from thymic maldescent [4]. Thymoma arising in ectopic thymic tissue, although a rare finding, is well documented. The most common location for ectopic thymomas is in the cervical region, accounting for almost 45% of all cases, this is followed by the lungs (20%), pleura (13%), pericardium (7%), thyroid (6%), middle/posterior mediastinum (4%) and other miscellaneous sites in decreasing order of frequency [5].

Patients presenting with mediastinal thymomas may be clinically asymptomatic (50-60%), or present with local symptoms (30-40%), systemic paraneoplastic disease syndromes (30-50%) [6]. Local symptoms, mainly non-specific chest pain, difficulty breathing, and cough, are the most common clinical presentations. MG is the most commonly associated disease when there is a systemic paraneoplastic disease [6], a finding noted in the reported case, too. 

The pathophysiology of MG in thymoma is attributed to the production of anti-acetylcholine receptor antibodies by the thymic cells, thereby resulting in MG [7]. Currently, thymectomy is strongly recommended in cases of thymomatous MG and non-thymomatous generalised MG with positive acetylcholine receptor antibody (AChR-Ab), to improve long-term clinical outcomes [8].

FNAC is often the initial line of investigation for a mediastinal swelling in a resource-poor setting. Ultrasound-guided or computed tomography-based FNAC is a relatively safe, cost-effective, and minimally invasive procedure in cases presenting as mediastinal masses, and may obviate the need for an invasive modality of investigation [9]. However, the WHO type B1/B2 thymomas, which are the lymphocyte-rich variants, are often mistaken for lymphoma by FNAC study. The major pitfall in differentiating lymphocyte-rich B1/B2 thymoma from lymphoma is primarily attributed to the overwhelming population of lymphoid cells, which may obscure the epithelial cells, cytomorphologically mimicking a lymphoid neoplasm. A few morphological and immunohistochemical highlights, which can be of immense importance in proper categorisation and ruling out various differential diagnoses of a thymic neoplasm arising in an ectopic site, are noted in Table 1.

**Table 1:** Key histomorphological and immunohistochemistry findings differentiating type B1/B2 thymoma, lymphoma, and thymic carcinoma.

Differential Diagnosis	Key Histomorphology	Immunohistochemistry Findings
Type B1/B2 Thymoma	Neoplastic epithelial cells admixed with immature T lymphocytes B1: Lymphocyte predominant, epithelial cells lesser in number B2: More prominent population of epithelial cells Low mitosis, rare to absent necrosis	Epithelial cells: AE1/AE3, CK19, Pan-CK, p40, p63: Positive Immature T-lymphocytes: TdT, CD1a, CD99, CD3 (confirms T-cell lineage): Positive Ki-67 labeling index: Low in epithelial cells, high in immature T-lymphocytes.
Lymphoma	Monotonous population of lymphoid cells. No epithelial cells. High mitotic activity may be noted.	Epithelial cell markers: Negative. Immature T-cell markers: Positive in case of T-lymphoblastic leukemia/lymphoma. Other markers: Lineage specific (CD3-T-cells, CD 20-B-cell) Further markers based on specific morphology
Thymic Carcinoma	Sheets/nests of atypical epithelial cells, lymphocytes are scant.	Epithelial cell marker: Strong diffuse positivity Immature T-cell marker: Usually negative Other markers: CD5, CD117,GLUT-1, MUC-1: Variable positivity Ki-67 labeling index:: High in neoplastic cells

Based on the presence of abundant lymphocytes, in our case, too, lymphoma was a strong contender in our initial FNAC examinations. However, cytologically neoplastic cells in cases of lymphoblastic lymphoma are larger with irregular nuclear contours compared to the smaller, more uniform cells in Type B thymoma [10]. In such cases, it is prudent to consider the possibility of an ectopic thymoma too and proceed accordingly. Cytomorphological findings in various types of thymoma, which may be useful inthe proper categorization of such neoplasms, are highlighted in Table 2.

**Table 2:** Cytomorphological highlights of various types of thymoma.

Types of Thymoma	Cytomorphological Findings
Type A	Epithelial cells: Abundant cohesive fragments of spindle/oval shaped, bland nuclei, fine chromatin, and inconspicuous nucleoli. Lymphocytes scant to absent.
Type B1	Abundant small mature lymphocytes, scant epithelial cells
Type B2	Abundant epithelial cells in cohesive clusters or syncytial sheets, scant small mature lymphocytes
Type B3	Predominantly mild-to-moderately atypical epithelial cell population, fewer lymphocytes compared to B2; may mimic Squamous cell carcinoma.
Type AB	Areas resembling type A (spindle cell rich) and type B (lymphocyte-rich area) noted.
Micronodular thymoma with lymphoid stroma (MNT)	Micronodular clusters of scant spindle/oval epithelial cells, against an abundant lymphoid background; may mimic lymphoma.
Metaplastic thymoma	Biphasic pattern: epithelial/spindle-shaped mesenchymal-like cells, cohesive epithelial nests.

Cell block preparation and examination is one such important, inexpensive technique that can be carried out in aspirated material and can be of immense help in making a correct diagnosis and eventually leading to better patient management [9]. Based on the abnormal location, an incisional biopsy and, eventually, resection of the para-tracheal mass were undertaken, which was subjected to histomorphology and immunohistochemical studies, which further substantiated the finding of Type B2 thymoma as noted in the cell block examination. This confirms the utility of cell blocks in making an appropriate diagnosis without subjecting the patient to an invasive procedure and yet establishing the correct diagnosis. 

Association of MG with ectopic thymoma is a rare finding; this case report highlights the rarity and also the potential of an ectopic thymoma to mimic a wide range of neoplastic conditions clinically and radiologically. It also highlights the clinician’s and pathologist’s dilemma in such cases. The report emphasizes that the use of relatively inexpensive and minimally invasive procedures like FNAC, eventually leading to cell block examination, can be an alternative to incisional biopsy in such rare cases.

## Conclusions

Thymoma arising in ectopic thymic tissue is a rare finding, and even rarer is the occurrence of MG in such patients. A clinico-pathological dilemma is unavoidable in such cases, but a strong clinical suspicion backed by laboratory findings can be of immense importance in the diagnosis and eventual management of these cases. 
